# Role of N^6^-Methyladenosine RNA Modification in Cardiovascular Disease

**DOI:** 10.3389/fcvm.2021.659628

**Published:** 2021-05-07

**Authors:** Dandan Song, Jianhua Hou, Junduo Wu, Junnan Wang

**Affiliations:** ^1^Department of Clinical Laboratory, Second Hospital of Jilin University, Changchun, China; ^2^State Key Laboratory of Inorganic Synthesis and Preparative Chemistry, College of Chemistry, Jilin University, Changchun, China; ^3^Department of Orthodontics, Hospital of Stomatology, Jilin University, Changchun, China; ^4^Department of Cardiology, Second Hospital of Jilin University, Changchun, China

**Keywords:** M6A, mRNAs, non-coding RNAs, CVD, modification

## Abstract

Despite treatments being improved and many risk factors being identified, cardiovascular disease (CVD) is still a leading cause of mortality and disability worldwide. N^6^-methyladenosine (m^6^A) is the most common, abundant, and conserved internal modification in RNAs and plays an important role in the development of CVD. Many studies have shown that aabnormal m^6^A modifications of coding RNAs are involved in the development of CVD. In addition, non-coding RNAs (ncRNAs) exert post-transcriptional regulation in many diseases including CVD. Although ncRNAs have also been found to be modified by m^6^A, the studies on m^6^A modifications of ncRNAs in CVD are currently lacking. In this review, we summarized the recent progress in understanding m^6^A modifications in the context of coding RNAs and ncRNAs, as well as their regulatory roles in CVD.

## Introduction

Cardiovascular disease (CVD) is a leading cause of mortality and disability worldwide despite recent improvements in health care, with many risk factors identified (1). Therefore, the mechanisms underlying CVD development remain to be elucidated. Recently, abnormal modifications in RNA have been identified in CVD and have attracted attention to our understanding of the mechanism underlying CVD development (2).

Currently, over 100 chemical modifications of RNA have been identified. Among them, N^6^-methyladenosine (m^6^A) is the most common conserved internal modification in RNA and is activated by the m^6^A methyltransferases (m^6^A writers), reversed by m^6^A demethylases (m^6^A erasers), and recognized by m^6^A-binding proteins (m^6^A readers) (3). m^6^A is enriched in the 3′ untranslated regions (3′-UTRs), stop codons, internal long exons, and consensus sequence RRACH (where R: A or G and H: A, C, or U), thus affecting mRNA splicing, export, translation, and decay (4). m^6^A modification is also present in the 5′ cap, which is required for RNA stability or degradation (5). m^6^A modification accounts for ~50% of the mRNA modifications in mammals (6). In addition to mRNAs, the m^6^A modification is also found in non-coding RNAs (nc RNAs) including micro RNAs (miRNAs), long non-coding RNAs (lnc RNAs), and circular RNAs (circ RNAs), which have been found to regulate transcription in many diseases including CVD (6–9).

Abnormal m^6^A RNA modifications have been found in the CVD risk conditions and to regulate the CVD development. However, research regarding the underlying mechanism is still lacking. In this review, we summarize the recent progress on the m^6^A modifications of mRNAs and ncRNAs, as well as their regulatory roles in CVD.

## Biology of m^6^A RNA Modification

**Figure 1:**
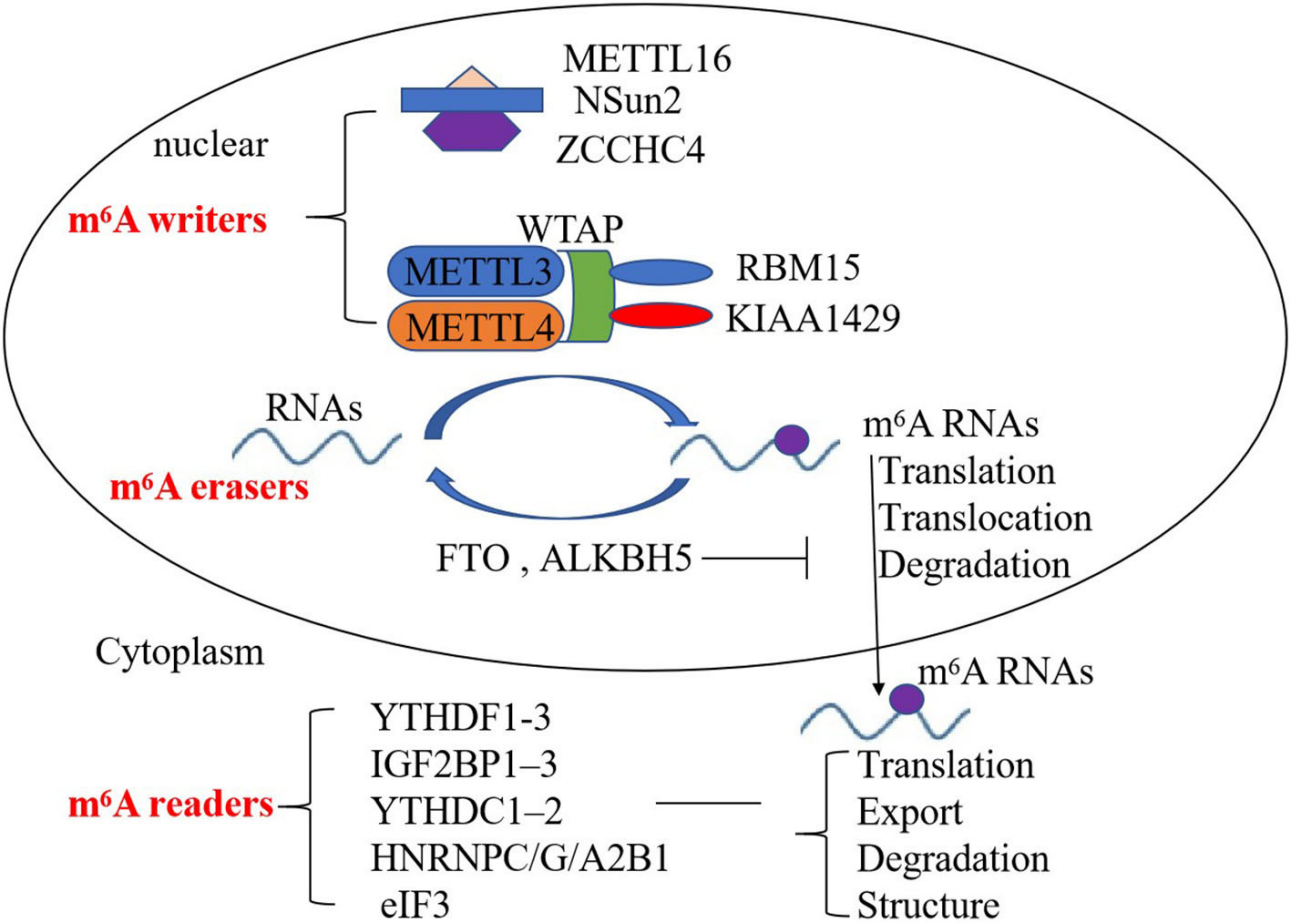
Mechanism of the deposition of m^6^A modification on mRNA and non-coding RNAs. m^6^A writers, erasers, and readers regulate the deposition of m^6^A modification and affect the translocation, translation, stability, degradation, and structure of RNA.

The m^6^A RNA modification is dynamically regulated by diverse functional proteins, including m^6^A methyltransferases, demethylases, and others (m^6^A writers, erasers, and readers, respectively). m^6^A writers include methyltransferase-like 3/14/16 (METTL3, METTL14, METTL16), Wilm's tumor-associated protein (WTAP), RNA-binding motif protein 15 (RBM15)—and its paralog RBM15B—and KIAA1429, NOP2/Sun RNA methyltransferase 2 (NSun2), and zinc finger CCHC domain-containing protein 4 (ZCCHC4) (10–13). METTL3 and METTL14 are the core components of the m^6^A writers; WTAP, RBM15, and KIAA1429 are also important components of the m^6^A methylase complex to enhance methyltransferase activity (10, 11). METTL16, NOP2/Sun RNA methyltransferase 2 (NSun2), and zinc finger CCHC domain-containing protein 4 (ZCCHC4) are other components of the m^6^A methylase complex, which are indispensable for m^6^A deposition (12, 13). m^6^A erasers consist of fat mass and obesity-associated protein (FTO) and AlkB family member 5 (ALKBH5) and can mediate m^6^A demethylation (14). m^6^A readers include YT521-B homology (YTH) domain family proteins (YTHDF1-3), insulin-like growth factor 2 mRNA-binding protein (IGF2BP), YTH domain-containing proteins (YTHDC), heterogeneous nuclear ribonucleoprotein (HNRNP), and eukaryotic translation initiation factor 3 (eIF3), which are also involved in m^6^A modification (15–19). YTHDC1 can also regulate target gene transcription (16, 17). YTHDF1 can bind to the 3′UTRs and the stop codon of m^6^A-containing RNAs, and interact with eIF3 to initiate translation (18). IGF2BPs, HNRNPC, and HNRNPG affect the stability, storage, the structure of RNA (18, 19). Therefore, m^6^A writers, erasers, and readers affect the translation, export, degradation, and structure of RNAs to regulate the development of many diseases (Figure 1).

### m^6^A Modification of mRNAs

m^6^A methylation is the most prevalent internal post-transcriptional modification of mammalian mRNA and can affect mRNA splicing, translocation, translation, stability, and structure (20). m^6^A modification frequently occurs in the introns of pre-mRNAs and promotes the nuclear export of mRNAs and facilitates mRNA transcription in the cytoplasm (21). m^6^A writer, METTL3 accelerates mRNA translocation from the nucleus to the cytoplasm and enhances translation of target mRNAs (22, 23). Moreover, METTL3 and METTL14 also reduce mRNA stability and promote mRNA degradation efficiency (5). In contrast, m^6^A eraser, ALKBH5 inhibits mRNA export and stability (14, 24). m^6^A readers are also found to regulate mRNA in many ways. For example, YTHDF1, YTHDF3, and IGF2BP1/2/3 can drive mRNA translation and promote translation efficiency (25, 26). Furthermore, IGF2BP also can enhance the stability of mRNA by binding to mRNA-stabilizing proteins such as human antigen R (HuR) (27). HuR is an RNA binding protein and can increase RNA stability (28). However, m6A modification can interact with HuR and inhibit its ability of enhancing RNA stability (28). YTHDF2 can promote the degradation of m^6^A-containing mRNA by recruiting RNA-degrading enzymes or adaptor proteins CCR4/NOT or HRSP12-RNase (29). Similarly, HNRNPC and HNRNPG can recognize specific sites on mRNA, thereby altering the structure of mRNAs (19). m^6^A writers, erasers, and readers affect mRNA expression in many aspects.

### m^6^A Modification of nc RNAs

Nc RNAs exert post-transcriptional regulation in many diseases and mainly include miRNAs, lnc RNAs, and circ RNAs (30). miRNAs are small nc RNA molecules ~22 nucleotides in length that bind with the 3′-UTR of mRNA to post-transcriptionally regulate genes (31). Lnc RNAs are ncRNAs that are longer than 200 nucleotides in length and circ RNAs are a specific class of ncRNA that form a covalently closed loop, and they interfere with gene expression and signaling pathways at various stages, such as the sponging of miRNAs (32, 33). Recently, many studies showed that miRNAs, lnc RNAs, and circ RNAs are modified by m^6^A (28, 34–45).

The m^6^A writers, METTL3 and METTL14 affect miRNA maturation by interacting with DiGeorge critical region 8 (DGCR8), which can bind to pri-miRNAs and promote miRNA maturation (34, 35). HuR is also found to increase miRNA stability by interfering with the binding of miRNAs to the Ago complex (28). The m^6^A eraser, FTO can enhance the stability of hsa-miR-6505-5p, hsa-miR-651-5p, and hsa-miR-493-5p, and reduce the stability of hsa-miR-7-5p, hsa-miR-92a-1-5p, and hsa-miR-6769a-3p, but the underlying molecular mechanism is not clear (36). While m6A modifies miRNA, miRNAs can also target m6A independently. For example, the miRNA let-7g binds to the 3′-UTR of METTL3 mRNA to inhibit its expression (37). Similarly, miR-145 targets the mRNA encoding YTHDF2 and inhibits YTHDF2 expression, which can stabilize m^6^A-modified mRNAs (38). Therefore, there is crosstalk of m^6^A modification with miRNAs.

Many m6A-methylated lnc RNA transcripts have been identified in mouse transcriptome (46). For example, METTL3 can increase the nuclear accumulation of lnc RNA RP11 to enhance its expression in colon cancer (39). METTL16 can methylate 68 lnc RNAs in human embryonic kidney 293 cells (40). By contrast, the m^6^A eraser, ALKBH5, can demethylate lnc RNA KCNK15-AS1 and nuclear paraspeckle assembly transcript 1 (NEAT1) (41). ALKBH5 was also found to reduce the m^6^A level and increase the stability of lnc RNA growth arrest-specific 5 (GAS5) (42). The m^6^A readers, YTHDF2 and YTHDF3 were found to promote the degradation of GAS5 (42, 43). The m^6^A reader IGF2BP2 interacts with the lnc RNA DANCR and stabilizes DANCR RNA (44). In addition, YTHDC1 and YTHDF2 are found to regulate the export and stability of circ RNAs (29, 45). Thus, the m^6^A modification exerts the regulatory effect through regulating the expressions of lnc RNAs and circ RNAs.

## m^6^A RNA Modification in Cardiovascular Risk Conditions

Many risk factors of CVD such as hyperlipidaemia, diabetes, and inflammation have been identified, but their molecular mechanisms in regulating CVD are still investigated (47–49). Recently, it has been found that m^6^A RNA methylations are dysregulated in risk conditions, and involved in the pathology of CVD (50, 51). These findings may provide insight into the molecular mechanisms underlying CVD development.

Hyperlipidaemia and obesity are risk factors for CVD development, and m^6^A functional enzymes are dysregulated and involved in lipid metabolism (47, 50). Oscillations in mRNA m^6^A methylation in the murine liver depend on a functional circadian clock, which is essential for lipid metabolic homeostasis (52). m^6^A methylation of peroxisome proliferator-activated receptor α (PPaRα) mRNA that codes for a nuclear receptor can accelerate lipid metabolism (22). m^6^A modification of PPaRα mRNA was decreased by METTL3 knockdown, causing the reduction of cellular lipid accumulation (53). In addition, m^6^A erasers and readers are also involved in lipid metabolism. FTO facilitates the adipogenesis of 3T3-L1 cells by interacting with YTHDF2 to maintain FTO-induced m^6^A demethylation (54). Consistently, FTO inhibition suppresses adipogenesis through an m^6^A-YTHDF2-dependent mechanism (54, 55). YTHDF2 is found to promote lipid accumulation by directly binding to the m^6^A modification site to promote the translation of 6-phosphogluconate dehydrogenase, which can increase the level of cholesterol FAM134B (56). Similarly, YTHDF1 is also found to promote adipogenesis in intramuscular preadipocytes by enhancing the translation of mitochondrial carrier homolog 2, which limited energy utilization and promoted diet-induced obesity (57). These evidences show that m^6^A writers, erasers, and readers can regulate lipid metabolism genes, which are involved in the development of CVD.

Diabetes is another risk factor positively correlated with the incidence of CVD (48). In patients with type 2 diabetes (T2D), m6A levels were reduced, while the mRNA levels of FTO, METTL3, METTL14, WTAP were significantly elevated and involved in the pathogenesis of diabetes (51). However, high glucose was found to enhance FTO levels in HepG2 cells (58). FTO can participate in glucose and insulin metabolism by inducing the expression of forkhead box O1 (FOXO1), glucose-6-phosphatase catalytic subunit, and diacylglycerol O-acyltransferase 2 mRNA (51). METTL3 and METTL14 were found to regulate insulin secretion in human β-cells. METTL14 inhibition can inhibit β-cell proliferation and promote insulin dysregulation (58). These findings indicate that FTO, METTL3, and METTL14 play important roles in the development of diabetes or CVD by regulating glucose metabolism and insulin secretion.

Inflammation was found in all phenomena associated with CVD including vascular and cardiac dysfunction (49). For example, M1-type macrophage-mediated inflammation plays an important role in the development of atherosclerosis (59). METTL3 expression is increased in M1-type macrophages and can directly methylate the mRNA of signal transducer and activator of transcription 1 to increase its expression (60). METTL3 can also promote the activation of dendritic cells by activating toll-like receptor 4 (TLR4)/nuclear factor-κB (NF-κB) signaling and increasing the expression of CD40, CD80, and IL-12 (61). METTL3 also can stimulate T cells and promote their differentiation (61, 62). METTL14 was found to promote an inflammatory response in endothelial cell (EC) and atherosclerotic plaque formation by interacting with FOXO1 and mediating its m^6^A modification (63). FOXO1 is an important transcription factor that acts directly on the promoter regions of VCAM-1 and ICAM-1 to promote their transcription (63). This evidence indicates that METTL3 and METTL14 can promote inflammation to regulate the development of CVD.

## m^6^A RNA Methylation in CVDs

**Figure 2:**
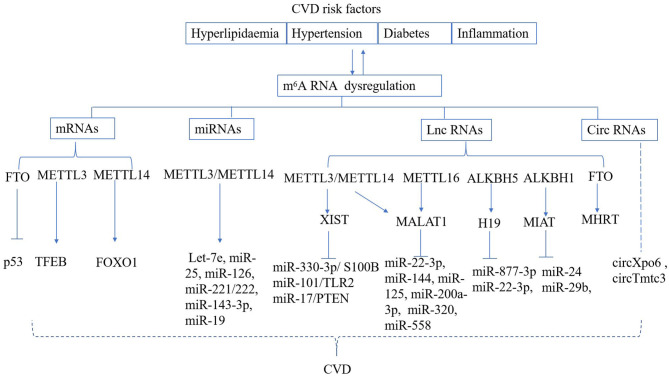
Regulation of m^6^A RNA modification in CVD. CVD risk factors can result in deregulated m^6^A deposition on mRNA, miRNA, lnc RNA, circ RNA, which are known to play a role in the development of cardiac and vascular disease.

CVD risk factors, such as hyperlipidaemia, hyperglycaemia, and inflammation can lead to vascular dysfunction, which ultimately results in cardiomyocyte ischemic injury and myocardial infarction (MI) (64, 65). The fibroblasts are activated and extracellular matrix (ECM) components are over-produced after MI; these compensate for cardiomyocyte loss and maintain the structural integrity of the ECM (66). Excessive cardiac remodeling and fibrosis following the cardiac injury can cause cardiomyocyte hypertrophy, which ultimately leads to heart failure (67). Dysregulated m^6^A RNA methylation has also been found to be responsible for vascular or cardiac dysfunction (Figure 2).

### m^6^A Modification of mRNAs in CVDs

METTL3 and FTO have been found to regulate vascular or cardiac dysfunction under stress conditions (68). METTL3 induced by hypoxic stress can promote the differentiation of adipose-derived stem cells into vascular smooth muscle cells (VSMCs) by increasing the expression of paracrine factors, including VEGF, and TGF-β (69). Similarly, METTL3 was also found to promote the differentiation of mouse embryonic stem cells into cardiomyocytes (70). In addition, METTL3 promoted the apoptosis of hypoxia and reperfusion (H/R)-treated cardiomyocytes by regulating the expression of transcription factor EB, which is a master regulator of lysosomal biogenesis and autophagy genes (71). Moreover, METTL3 promotes cardiac remodeling and hypertrophy by catalyzing the m^6^A methylation of certain subsets of mRNAs (70). In contrast, METTL3 knockout hearts develop maladaptive eccentric remodeling and cardiac functional defects with aging and rapid progressive dysfunction following acute pressure-overload stress (72). Cardiac FTO expression is decreased in cardiomyocytes under conditions of hypoxia, ischemia, and heart failure (73). It has been observed that FTO overexpression attenuates hypoxia-induced cardiomyocyte dysfunction and restores calcium handling and sarcomere dynamics (73). FTO has been shown to attenuate ischemia-induced cardiac remodeling and improve cardiac contractility by demethylating the m^6^A modifications of p53, thereby inhibiting the expression of p53 (74, 75). Thus, m^6^A writers, erasers, and readers can regulate the developments of vascular and cardiac diseases *via* the methylation of target mRNAs.

### m^6^A-Methylated miRNAs in CVD

miRNAs are a determinant of cardiovascular pathology and could be modified by m^6^A (31). For example, m^6^A modification and METTL14 are significantly up-regulated in atherosclerotic vascular endothelial cells and promote their proliferation (76). The underlying mechanism is that the METTL14 inhibits the expression of pri-miR-19a but increases the expression of mature miR-19a by binding to DGCR8 (76). Similarly, METTL3 homolog, mRNA adenosine methylase (MTA) can accumulate primary pri-miRNAs but inhibits the expression of mature miRNAs In Arabidopsis (77). In addition, many miRNAs are found to be mediated the deposition of m^6^A modification by METTL3 or METTL14, and some of them play important roles in CVD development (78). For example, METTL3 affect the stability of Let-7e, miR-25, miR-126, miR-221/222, and miR-143-3p (78). METTL14 modulates the primary processing of miR-126 and miR-375 by interacting with DGCR8 in hepatocellular carcinoma or colon cancer, respectively (34, 79). Let-7, miR-126, miR-221/222, and miR-143-3p are key vascular biology players that are involved in the development of atherosclerosis and angiogenesis *via* their effects on ECs and VSMCs (80–85). Let-7, miR-25, and miR-375 play an important role in the development of cardiac diseases, including arrhythmia, dilated cardiomyopathy, MI, cardiac hypertrophy, fibrosis, and heart failure by regulating apoptosis, autophagy, oxidative stress, inflammation, and calcium handling (80, 86, 87). Those pieces of evidence indicate that m^6^A modifications are involved in the development of CVD by affecting the expressions of miRNAs.

### m^6^A Methylation of lnc RNAs and Circ RNAs in CVDs

Similar to miRNAs, lnc RNAs and circ RNAs have been thoroughly investigated in the context of CVD and have recently been found to be m^6^A-methylated (32, 88). For example, the m^6^A modification is enriched on lnc RNA 1281 and the m^6^A modification of lnc RNA 1281 affects the differentiation of embryonic stem cells (ESC) *via* sponging Let-7, which has been reported to play an important role in the cardiovascular differentiation of ESCs and the development of CVD (80, 89).

The lnc RNA H19 is highly expressed in human atherosclerotic lesions and promotes the development of atherosclerosis by regulating the mitogen-activated protein kinase and NF-kB signaling pathways (90). Additionally, H19 ameliorates ischemia-reperfusion (I/R)-induced myocardial apoptosis or MI-induced myocardial injury by sponging miR-877-3p or miR-22-3p, respectively (91, 92). In H9c2 cells with H2O2-induced senescence, H/R enhanced the level of m^6^A methylation and increased the expression of lnc RNA H19 by upregulating ALKBH5 (93). Therefore, the m^6^A modification of H19 is involved in the development of CVD.

Lnc RNA myocardial infarction associated transcript (MIAT) is also found to inhibit EC proliferation, migration, and tube formation in diabetes *via* the sponging of miR-29b (94). MIAT levels were also increased in MI and deregulated some fibrosis-related regulators by sponging miR-24 and increasing the expression of furin and TGF-β1 (95). Similarly, the MIAT levels increase in response to hypoxia, and MIAT is involved in cardiac interstitial fibrosis (96). Oxidized low-density lipoprotein (ox-LDL)-induced m^6^A demethylation was found to facilitate the binding of HIF1α to the ALKBH1-demethylated MIAT promoter and the transactivation of MIAT, indicating that MIAT is a target gene of ALKBH1-related m^6^A methylation (97).

Lnc RNA X-inactive specific transcript (XIST) was reported to play an important role in CVD development and is highly m^6^A-methylated (3). XIST was highly expressed in human thoracic aortic dissection and promoted the apoptosis of VSMCs by sponging miR-17 (98). Consistently miR-17 was reported to promote mitochondria-dependent apoptosis by targeting at phosphatase and tensin homolog deleted on chromosome 10 (PTEN) (98). In addition, XIST inhibited myocardial cell proliferation by sponging miR-130a-3p, which targets phosphodiesterase 4D (99). XIST was also found to promote phenylephrine-induced cardiac hypertrophy *via* the miR-330-3p/S100B and miR-101/TLR2 axis (100, 101). The METTL3/METTL14 complex deposited the 78 m^6^A-methylation on XIST RNA by interacting with the MACOM complex, comprising WTAP, VIRMA, and RBM15 proteins, and inhibited the expression of XIST (102). YTHDC1 and YTHDF2 bind to XIST and mediate its degradation (102). This evidence indicated that the m^6^A modification of XIST might regulate the development of CVD.

Metastasis-associated lung adenocarcinoma transcript 1 (MALAT1) regulates the development of CVD and contains several m^6^A motifs (103, 104). MALAT1 protects against endothelial injury induced by ox-LDL, hyperglycaemia, and oxidative stress *via* the sponging of miR-22-3p or activation of nuclear factor erythroid 2 (105, 106). MALAT1 levels were increased in the serum and myocardial tissue of AMI and promoted cardiomyocyte apoptosis or myocardial tissue injury induced by hypoxia, H/R, or I/R by targeting miR-144, miR-125, miR-200a-3p, or miR-320, respectively (107–110). However, it was also found that MALAT1 inhibits isoproterenol-induced cardiomyocyte apoptosis by sponging miR-558 (111). Additionally, MALAT1 promoted angiotensin II-induced cardiac fibrosis by sponging miR-145, thereby enhancing target growth factor-β1 activity (112). Recently, the m^6^A-deposition sites of MALAT1 have been identified. For example, m^6^A modification at the A2577 or A2515 site of MALAT1 can destabilize the RNA hairpin, release the poly(U) tract, and increase binding with HNRNPC or HNRNPG, respectively (103, 104). METTL16 specifically binds to the 3′-end of a triple-helix and enhances the stability of MALAT1 transcripts (113). This evidence indicates that MALAT1 could be m^6^A modified to regulate the development of CVD.

The levels of GAS5 were increased in atherosclerotic rats and aggravated ox-LDL-induced inflammation by inhibiting the expression of miR-221 or miR-135a (114, 115). GAS5 was also found to accelerate myocardial I/R injury by sponging miR-532-5p (116). In contrast, other studies showed that GAS5 could attenuate homocysteine-induced cardiac microvascular ECs by inhibiting miR-33a-5p and reverse cardiac apoptosis and fibrosis *via* the inhibition of semaphorin-3A or miR-21 expression, respectively (117–119). The m^6^A modifications of GAS5 have also been identified. ALKBH5 reduced the m^6^A level and increased the stability of GAS5. m^6^A induced GAS5 RNA degradation in a YTHDF2-dependent manner (42). Knockdown of YTHDF3 was also found to prolong the degradation of GAS5. This evidence indicates that m^6^A-deposited GAS5 might be involved in the development of CVD (42, 43).

Lnc RNA, Myheart (MHRT), plays an important role in cardiac disease. MHRT protects against the H_2_O_2_ or H/R -induced apoptosis of cardiomyocytes (120). In addition, MHRT is found to regulate cardiac hypertrophy and is associated with the outcome of heart failure (121, 122). Over-expression of FTO protects against H/R-induced apoptosis of myocardial cells by regulating m^6^A modification of MHRT, indicating that m^6^A modification of MHRT participates in the development of cardiac disease (123).

Certain circ RNAs, such as circXpo6 and circTmtc3, have also demonstratedm6A-methylation in the lungs of rats with hypoxia-induced pulmonary hypertension, as well as in pulmonary artery smooth muscle cells, and, finally, in pulmonary arterial ECs exposed to hypoxia. This suggests that m6A-methylated circXpo6 and circTmtc3 might be involved in the development of CVD (124). However, the role of m^6^A-methylated circ RNAs in the development of CVD requires further study.

## Modulation of m^6^A for CVD Treatment

Modulation of m^6^A could be a strategy for CVD treatment. For example, silencing of METTL reduced I/R-induced cardiac injury and H/R-induced apoptosis of cardiomyocytes by inducing autophagy (71). Moreover, METTL3 inhibition reduced cardiomyocyte remodeling under the hypertrophic stimuli (125). Similarly, inhibition of METTL14 was found to decrease the calcification and enhance the vascular repair function (126). It was favored that inhibition of METTL14 inhibited the proliferation of atherosclerotic vascular endothelial cells by affecting the expression of miR-19 (76). Over-expression of FTO by adeno-associated virus serotype 9 (AAV9) significantly prevented the formation of atherosclerotic plaques by reducing total cholesterol (127). Furthermore, FTO over-expression significantly improved cardiac function by reducing fibrosis and increasing angiogenesis at the chronic stage of post-myocardial infarction (73). Moreover, the protective effect of FTO in cardiac disease is associated with the regulation of m^6^A modification of MHRT (123).

Non-coding RNAs also can regulate m^6^A Micro RNAs such as miR-33a and miR-4429 were found to inhibit METTL3 in the field of tumor studies, indicating that those miRNAs might be as therapeutic agents for CVD (128, 129). In addition, lnc RNA H19 has been reported to protect against H2O2-induced H9c2 cell apoptosis by up-regulating ALKBH5 (93). Thus, ncRNAs might be used for the regulation of m^6^A and CVD treatment.

## Conclusion

CVD is a leading cause of death worldwide, but the underlying mechanism remains unknown. m^6^A is the most common, abundant, and conserved internal modification in RNAs, including mRNA and ncRNAs. In this review, we summarized the current research on m^6^A RNA modification on CVD risk conditions and development, which may help elucidate the molecular mechanism underlying CVD development. In addition, inhibition of MELL3/14 or over-expression of FTO could be used for the treatment of CVD. Notably, some ncRNAs also can regulate m^6^A modifications and could be therapeutic molecules for CVD, However, m^6^A modifications of ncRNAs in CVD require further study.

## Author Contributions

JWa designed the article. DS, JH, JWu, and JWa wrote and critically reviewed the manuscript. All authors contributed to the article and approved the submitted version.

## Conflict of Interest

The authors declare that the research was conducted in the absence of any commercial or financial relationships that could be construed as a potential conflict of interest.

## Abbreviations

CVD: Cardiovascular disease
m^6^A,N^6^: methyladenosine
3′-UTRs: 3′ untranslated regions
miRNAs: micro RNAs
lnc RNAs: long non-coding RNAs
circ RNAs: circular RNAs
METTL3: methyltransferase-like 3
METTL14: methyltransferase-like 14
WTAP: Wilm's tumor-associated protein
RBM15: RNA-binding motif protein 15
IGF2BP: insulin-like growth factor 2 mRNA-binding protein
HNRNP: heterogeneous nuclear ribonucleoprotein
NSun2: NOP2/Sun RNA methyltransferase 2
ZCCHC4: zinc finger CCHC domain-containing protein 4
FTO: fat mass and obesity-associated protein
ALKBH5: AlkB family member 5
YTH: YT521-B homology
YTHDF: YTH domain family proteins
YTHDC: YTH domain-containing proteins
eIF3: eukaryotic translation initiation factor 3
DGCR8: DiGeorge Critical Region 8
T2D: type 2 diabetes
FOXO1: forkhead box O1
H/R: hypoxia and reperfusion
MI: myocardial infarction
ox-LDL: oxidized low-density lipoprotein
MALAT1: metastasis-associated lung adenocarcinoma transcript 1
EC: endothelial cell
VSMCs: vascular smooth muscle cells
XIST: X-inactive specific transcript
ESC: embryonic stem cells
I/R: ischemia-reperfusion
GAS5: growth arrest-specific 5
MIAT: myocardial infarction associated transcript
TLR4: toll-like receptor 4
NF-κB: nuclear factor-κB
ECM: extracellular matrix.
